# Nitroglycerine-Induced Nitrate Tolerance Compromises Propofol Protection of the Endothelial Cells against TNF-****α****: The Role of PKC-****β****_2_ and NADPH Oxidase

**DOI:** 10.1155/2013/678484

**Published:** 2013-12-12

**Authors:** Shaoqing Lei, Wating Su, Huimin Liu, Jinjin Xu, Zhong-yuan Xia, Qing-jun Yang, Xin Qiao, Yun Du, Liangqing Zhang, Zhengyuan Xia

**Affiliations:** ^1^Department of Anesthesiology, Renmin Hospital of Wuhan University, Wuhan, Hubei 430060, China; ^2^Department of Anesthesiology, University of Hong Kong, Hong Kong; ^3^Department of Cardiac Surgery, Chongqing Zhongshan Hospital, Chongqing 400013, China; ^4^Department of Anesthesia, Chongqing Zhongshan Hospital, Chongqing 400013, China; ^5^Department of Anesthesiology, Affiliated Hospital of Guangdong Medical College, Zhanjiang, Guangdong 524001, China

## Abstract

Continuous treatment with organic nitrates causes nitrate tolerance and endothelial dysfunction, which is involved with protein kinase C (PKC) signal pathway and NADPH oxidase activation. We determined whether chronic administration with nitroglycerine compromises the protective effects of propofol against tumor necrosis factor (TNF-) induced toxicity in endothelial cells by PKC-**β**_2_ dependent NADPH oxidase activation. Primary cultured human umbilical vein endothelial cells were either treated or untreated with TNF-**α** (40 ng/mL) alone or in the presence of the specific PKC-**β**_2_ inhibitor CGP53353 (1 **μ**M)), nitroglycerine (10 **μ**M), propofol (100 **μ**M), propofol plus nitroglycerin, or CGP53353 plus nitroglycerine, respectively, for 24 hours. TNF-**α** increased the levels of superoxide, Nox (nitrate and nitrite), malondialdehyde, and nitrotyrosine production, accompanied by increased protein expression of p-PKC-**β**_2_, gP91phox, and endothelial cell apoptosis, whereas all these changes were further enhanced by nitroglycerine. CGP53353 and propofol, respectively, reduced TNF-**α** induced oxidative stress and cell toxicity. CGP53353 completely prevented TNF-**α** induced oxidative stress and cell toxicity in the presence or absence of nitroglycerine, while the protective effects of propofol were neutralized by nitroglycerine. It is concluded that nitroglycerine comprises the protective effects of propofol against TNF-**α** stimulation in endothelial cells, primarily through PKC-**β**_2_ dependent NADPH oxidase activation.

## 1. Introduction

Ischemic heart disease is a leading cause of death in many regions. The mortality of myocardial infarction remains significant despite advancement in surgical techniques and pharmacological therapies. Organic nitrates (such as nitroglycerin, L-arginine) are still useful drugs and have been widely used for the prevention and treatment of ischemic heart disease for more than 100 years [1, 2]. However, these drugs are also known to induce nitrate tolerance after prolonged, continuous, or high dose administration, which leads to the abolishment of clinical or hemodynamic response to organic nitrates [3] and subsequently induces endothelial dysfunction [4]. It has been reported that nitrate tolerance and endothelial dysfunction are associated with increased vascular production of reactive oxygen species (ROS) via mechanisms that involve increased protein kinase C (PKC) and NADPH oxidase activation, eNOS uncoupling in the vascular endothelium [4–6]. Interestingly, circulatory proapoptotic inflammatory cytokines (such as tumor necrosis factor (TNF-)*α*), which are increased during myocardial infraction and atherosclerosis, may promote the production of ROS subsequent to the induction of cardiomyocyte apoptosis and endothelial cells apoptosis [7]. A further study had shown TNF-*α* induced human endothelial cell apoptosis which involved the activation of PKC [8]. Despite these observations, whether or not organic nitrates aggravate TNF-*α* induced endothelial cell apoptosis and the underlying mechanisms by which PKC isoforms exert adverse effects in this pathology remain unclear.

Propofol, an anesthetic with demonstrated antioxidant properties [9], has shown protective effects in various models against ischemia-reperfusion injury [10–12]. We previously reported that propofol dose-dependently reduced TNF-*α* induced apoptosis in primary cultured human umbilical vein endothelial cells (HUVECs) [13]. Our further study showed that the supplementation of L-arginine exacerbated TNF-*α* induced cellular toxicity by enhancing oxidative stress and nitrative stress, which was neutralized by propofol treatment [14]. It is unknown, however, whether or not propofol achieves these effects via inhibition of PKC-*β*_2_, a PKC isoform that may play a major role in TNF-*α* induced human endothelial cell apoptosis [15]. Of interest, propofol has been shown to activate PKC-*α*, PKC-*δ*, PKC-*ε*, and PKC-*ξ* in cardiomyocytes [16–18], which may represent an important cellular mechanism of propofol-induced myocardial protection in the setting of ischemia-reperfusion injury. However, in all these studies, the effect of propofol on PKC-*β*_2_ has not been reported, nor has it been investigated in endothelial cells in the condition of nitrate tolerance. In the present study, we hypothesize that nitrate tolerance induced by organic nitrates comprises the protective effects of propofol against TNF-*α* induced toxicity in endothelial cells. Our data suggests that nitroglycerine supplementation promoted PKC-*β*_2_ activation in HUVECs subjected to TNF-*α* stimulation, which subsequently increased the activation of NADPH oxidase and compromised the protective effects of propofol against TNF-*α* induced damage.

## 2. Materials and Methods

### 2.1. Cell Culture

Primary cultured HUVECs will be prepared using established procedures as previously described [13]. Briefly, cells were digested from the umbilical vein with 0.1% collagenase I (w/v) at 37°C for 20 min, after which they were cultured in 0.1% (w/v) gelatin-coated flasks in Medium 199 supplemented with 10% fetal bovine serum, 15 mg/L ECGS, 2 mM glutamine, 100 units/mL penicillin, and 100 *μ*g/mL streptomycin in an atmosphere of 5% CO_2_ at 37°C. The medium was changed every 2-3 days until the ECs reached confluence. Cultured cells were identified as ECs by their morphology and the presence of the Factor VIII-related antigen was detected using an indirect immunocytochemistry method as described [19]. The purity of HUVECs in culture was higher than 95% and passages 2–4 were used in the research.

### 2.2. Experimental Conditions

When the cells were at 70% confluence, the cultured cells were then randomly divided into the following groups: cells were either not treated (control group, Con.) or treated with 40 ng/mL TNF-*α* (TNF-*α* group, T) alone or TNF-*α* in the presence of 1 *μ*M CGP53353 (CGP) (TNF-*α* + CGP group, T + C), 10 *μ*M nitroglycerine (NTG) (TNF-*α* + NTG group, T + N), 100 *μ*M propofol (TNF-*α* + propofol group, T + P), or NTG plus propofol (TNF-*α* + NTG + propofol group, T + N + P), and NTG plus CGP53353 (TNF-*α* + NTG + CGP53353 group, T + N + C), respectively, for 24 hours. In specific groups, cultured cells were pretreated with propofol for 30 min before other treatments. The concentration of TNF-*α* used to induce apoptosis in the present study was chosen on the basis of our previous studies [13], which demonstrated that TNF-*α* at the dose of 40 ng/mL could significantly induce ECs apoptosis. The concentration of NTG adopted is according to the studies [20, 21], which demonstrated that NTG at the dose of 10 *μ*M could induce nitrate tolerance. The choice of concentration of PKC-*β*_2_ inhibitor was based on that 1 *μ*M CGP53353 could selectively inhibit PKC-*β*_2_ activation in our previous study [22]. In our preliminary study, propofol at the dose of 100 *μ*M reversed TNF-*α* (40 ng/mL) induced cell injury but propofol at the dose of 100 *μ*M per se did not cause apparent apoptosis under the present experimental condition in the absence of TNF-*α* stimulation. Therefore, we chose the concentration of 100 *μ*M as the treatment dose of propofol for the further mechanistic study.

### 2.3. Determination of Cytotoxicity

Cytotoxicity will be assessed by measuring lactate dehydrogenase (LDH) (Jiancheng Co., Nanjing, China) release in the medium in addition to the measurement of cell viability using the 3-(4,5-Dimethylthiazol-2-yl)-2,5-diphenyltetrazolium bromide (MTT) (Sigma, St. Louis, MO, USA) assay according to the manufacturer's instructions.

### 2.4. Determination of Lipid Peroxidation

The content of malondialdehyde (MDA), which is a marker of lipid peroxidation, was measured to evaluate the oxidative injury of ECs. After homogenizing on ice in normal saline, the MDA levels of the supernatants of cell samples was determined by the thiobarbituric acid colorimetric method using MDA assay kit (Jiancheng Co., Nanjing, China) as described [23, 24]. The results were expressed as nanomole per milligram protein (nmol/mg protein).

### 2.5. Determination of the Levels of *NO*_*x*_, *O*_2_^−^, and Nitrotyrosine

Cultured cells were homogenized in ice-cold PBS and centrifuged at 3,000 g for 15 minutes at 4°C for supernatant collection. The supernatant protein concentration was determined via a Lowry assay kit (Bio-Rad, CA, USA). Concentrations of nitrites (NO_2_^−^) and nitrates (NO_3_^−^), the stable end products of nitric oxide (NO), were determined by the Griess reaction as previously described [13]. NOx levels were expressed as *μ*mol/L protein. Myocardial *O*_2_^−^ production was determined via lucigenin chemiluminescence method [25, 26]. The supernatant samples were loaded with dark-adapted lucigenin (5 *μ*M) and read in 96-well microplates by luminometer (GloMax, Promega). Light emission, expressed as mean light units (MLU)/min/100 *μ*g protein, was recorded for 5 minutes. Myocardial nitrotyrosine levels (*μ*g/mg protein) in the collected supernatant were determined by chemiluminescence detection via the Nitrotyrosine Assay Kit per manufacturer's protocol (Millipore, USA).

### 2.6. Detection of Apoptosis by Flow Cytometry

DNA fragments which are lost from apoptotic nuclei and nuclear DNA content can be easily measured by flow cytometry after nucleic acid staining with specific fluorochromes. Briefly, cells (1 × 10^6^) were harvested and processed as described [14]. Then the cells were performed to Annexin-V-fluos Staining and analyzed using a flow cytometer (Beckman Coulter, Brea, CA) according to manufacturer's protocol. Electronic compensation of the instrument is required to exclude overlapping of the two emission spectra. All measurements were performed in the same instrumental settings.

### 2.7. Western Blot Analysis

Cultured cells were homogenized in cell lysis buffer containing Tris-HCl (20 mM, pH 7.4), NaCl (150 mM), EDTA (l mM), EGTA (l mM), *β*-glycerolphosphate (1 mM), sodium pyrophosphate (2.5 mM), Triton X-100 (1%), PMSF (1 mM), DTT (l mM), leupeptin (1 *μ*g/mL), aprotinin (1 *μ*g/mL), and pepstatin (1 *μ*g/mL). The homogenate was centrifuged at 1,000 g for 10 min at 4°C to collect the supernatant as total protein preparations. Equal amounts of protein were combined with 5×SDS loading buffer, boiled for 5 min, then separated via 10% SDS-PAGE, and subsequently transferred to PVDF membrane for immunoblot analysis. The membranes were blocked in 5% no fat milk for 2 hours at room temperature and then incubated overnight at 4°C with primary antibodies against p-PKC-*β*_2_ (ser660) (1 : 1000, Cell Signaling Technology), PKC-*β*2 (1 : 1000, Cell Signaling Technology), and gP91phox (1 : 500, Santa Cruz Biotechnology). After being washed with TBST, the membranes were incubated with proper secondary horseradish peroxidase (HRP-)conjugated antibodies (1 : 5,000–1 : 10,000, Cell Signaling Technology) and developed with enhanced chemiluminescence reagent (GE Healthcare, USA). The membranes were subsequently reblotted for GAPDH (1 : 2,000, Cell Signaling Technology), and the results were normalized to GAPDH to correct for loading.

### 2.8. Statistical Analysis

All the data are expressed as mean ± S.E.M. Significance was evaluated by analysis of one-way variance (ANOVA) followed by Tukey's test. GraphPad Prism software program (GraphPad Software Inc., San Diego, CA, USA) was used for statistical analysis. *P* < 0.05 was considered statistically significant.

## 3. Results

### 3.1. Cell Viability and LDH Release

**Figure 1:**
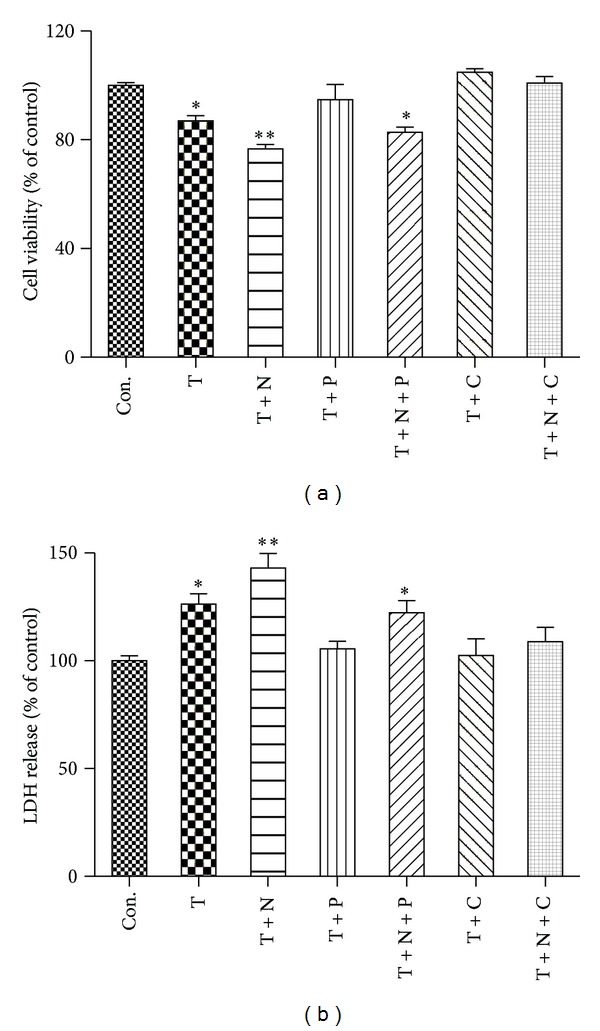
Cell viability (a) and LDH release (b). Primary cultured human umbilical vein endothelial cells (HUVECs) were either not treated (control, Con.) or treated with TNF-*α* (40 ng/mL) alone (T) or with TNF-*α* in the presence of nitroglycerine (10 *μ*M) (T + N), propofol (100 *μ*M) (T + P), CGP53353 (1 *μ*M) (T + C), propofol plus nitroglycerin (T + N + P), or CGP53353 plus nitroglycerine (T + N + C), respectively, for 24 h. All results are expressed as mean ± S.E.M., *n* = 7, *P < 0.05 compared with Con., T + N, T + P, T + C and T + N + C, **P < 0.01 compared with Con., T + P, T + C and T + N + C.

The cytotoxicity of the cultured endothelial cells was assessed by MTT assay and LDH release. As shown in Figure 1(a), cell viability was significantly reduced after TNF-*α* stimulation as compared with control, which was reversed by propofol treatment. The supplementation of nitroglycerine further exacerbated TNF-*α* induced reduction in cell viability. The treatment of propofol improved but not restored the viability of the cells subjected to TNF-*α* stimulation in the presence of nitroglycerine. By contrast, CGP53353, a selective inhibitor of PKC-*β*_2_, reversed the reduced cell viability induced by TNF-*α* with or without the presence of nitroglycerine.

Stimulation with TNF-*α* resulted in a significant increase in LDH release in the medium of cultured HUVECs (Figure 1(b)). Addition of nitroglycerine further increased TNF-*α* induced LDH release. Both propofol and CGP53353 significantly restored the TNF-*α* induced LDH release. By contrast, propofol reduced but not reversed the levels of LDH release in the presence of nitroglycerine.

### 3.2. Endothelial Cell Apoptosis

**Figure 2:**
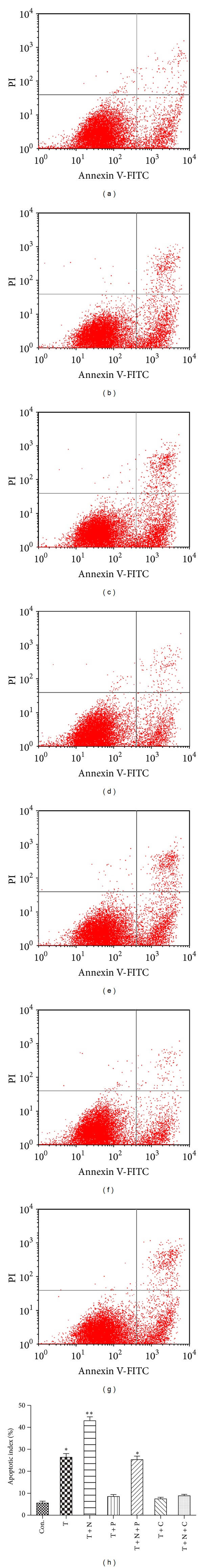
Representative figures of flow cytometry results (a)–(g) and rate of apoptotic cells measured by flow cytometry (h). Flow cytometric analysis was carried out as described in methods. Primary cultured human umbilical vein endothelial cells (HUVECs) were either not treated (control, Con.) or treated with TNF-*α* (40 ng/mL) alone (T) or with TNF-*α* in the presence of nitroglycerine (10 *μ*M) (T + N), propofol (100 *μ*M) (T + P), CGP53353 (1 *μ*M) (T + C), propofol plus nitroglycerin (T + N + P), or CGP53353 plus nitroglycerine (T + N + C), respectively, for 24 h. (a)–(g) Representatives of the C, T, T + N, T + P, T + N + P, T + C and T + N + C, respectively. All results are expressed as mean ± S.E.M., *n* = 7, *P < 0.05 compared with Con., T + N, T + P, T + C and T + N + C, **P < 0.01 compared with Con., T + P, T + C and T + N + C.

Stimulation of HUVECs with TNF-*α* resulted in a marked significant increase in apoptotic index (Figure 2). Nitroglycerine further increased TNF-*α* induced cell apoptotic death. On the other hand, CGP53353 and propofol significantly attenuated cell apoptosis induced by TNF-*α*. Propofol attenuated but not prevented the combination of nitroglycerine and TNF-*α* induced cell apoptotic death, which was profoundly decreased by the treatment of CGP53353. The patterns of apoptotic index results obtained from TUNEL staining were similar to those obtained by flow cytometry (data not shown).

### 3.3. Superoxide and MDA Production

**Figure 3:**
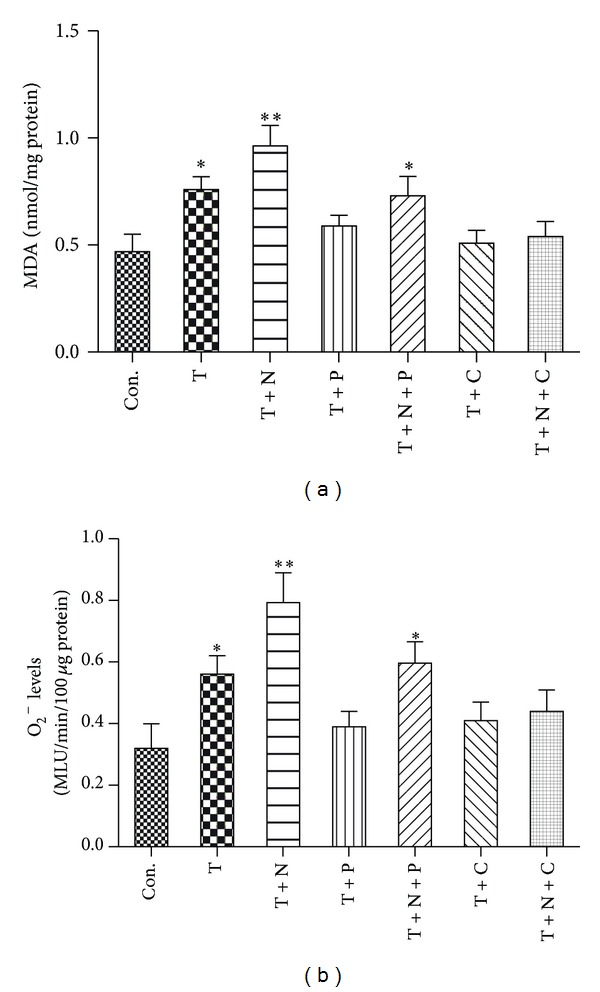
Effects of TNF-*α*, nitroglycerine, and CGP53353 on MDA (a) and superoxide production (b). Primary cultured human umbilical vein endothelial cells (HUVECs) were either not treated (control, Con.) or treated with TNF-*α* (40 ng/mL) alone (T) or with TNF-*α* in the presence of nitroglycerine (10 *μ*M) (T + N), propofol (100 *μ*M) (T + P), CGP53353 (1 *μ*M) (T + C), propofol plus nitroglycerin (T + N + P), or CGP53353 plus nitroglycerine (T + N + C), respectively, for 24 h. All results are expressed as mean ± S.E.M., *n* = 7, *P < 0.05 compared with Con., T + N, T + P, T + C and T + N + C, **P < 0.01 compared with Con., T + P, T + C and T + N + C.

As the production of ROS plays an important role in the development and progress of nitrate tolerance and endothelial dysfunction [4], we measured superoxide and MDA production, which is a marker of lipid peroxidation. As shown in Figure 3, the levels of superoxide and MDA were significantly increased in HUVECs subjected to TNF-*α* stimulation as compared to control group, which were prevented by the treatment of propofol or CGP53353. Addition of nitroglycerine further promoted the production of superoxide and MDA, which was neutralized by propofol treatment but reversed by CGP53353 treatment.

### 3.4. NOx and Nitrotyrosine Production

**Figure 4:**
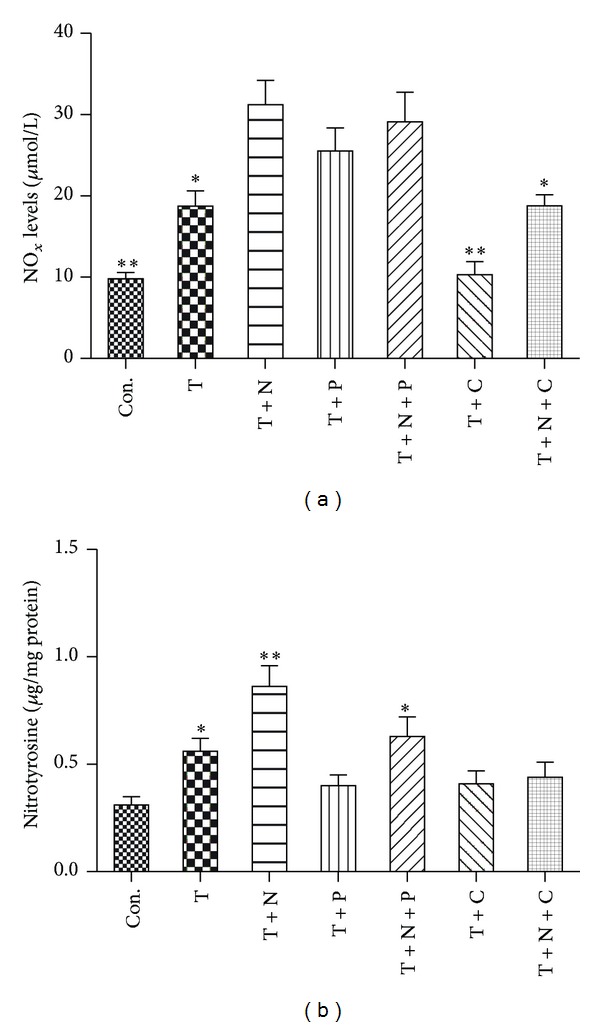
Effects of TNF-*α*, nitroglycerine and CGP53353 on NOx (a) and nitrotyrosine production (b). Primary cultured human umbilical vein endothelial cells (HUVECs) were either not treated (control, Con.) or treated with TNF-*α* (40 ng/mL) alone (T) or with TNF-*α* in the presence of nitroglycerine (10 *μ*M) (T + N), propofol (100 *μ*M) (T + P), CGP53353 (1 *μ*M) (T + C), propofol plus nitroglycerin (T + N + P), or CGP53353 plus nitroglycerine (T + N + C), respectively, for 24 h. All results are expressed as mean ± S.E.M., *n* = 7, (a) *P < 0.05 compared with Con., T + N, T + P, T + N + P and T + C, **P < 0.01 compared with T + N, T + P and T + N + P; (b) *P < 0.05 compared with Con., T + N, T + P, T + C and T + N + C, **P < 0.01 compared with Con., T + P, T + C and T + N + C.

We next determined the production of NOx and nitrotyrosine in HUVECs. Stimulation of TNF-*α* increased the levels of NOx and nitrotyrosine production, and nitroglycerine further increased their levels (Figure 4). Propofol treatment had no effects on NOx production in the cells subjected to TNF-*α* or combination with nitroglycerine stimulation, but significantly decreased TNF-*α* induced production of nitrotyrosine. By contrast, CGP53353 prevented TNF-*α* induced NOx production and nitroglycerine-mediated increase of NOx production and reversed TNF-*α* induced production of nitrotyrosine whether or not in the presence of nitroglycerine.

### 3.5. Protein Expression of p-PKC-*β*_2_ and gP91phox

**Figure 5:**
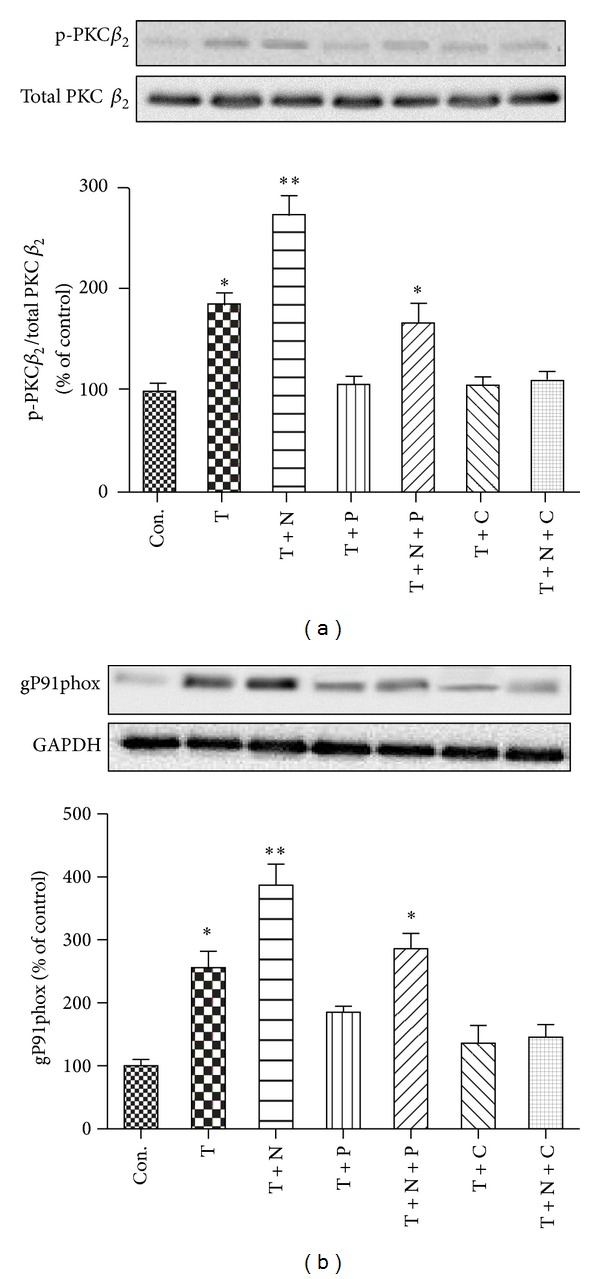
Effects of TNF-*α*, nitroglycerine, and CGP53353 on the protein expression of p-PKC-*β*_2_ (a) and gP91phox (b). Primary cultured human umbilical vein endothelial cells (HUVECs) were either not treated (control, Con.) or treated with TNF-*α* (40 ng/mL) alone (T) or with TNF-*α* in the presence of nitroglycerine (10 *μ*M) (T + N), propofol (100 *μ*M) (T + P), CGP53353 (1 *μ*M) (T + C), propofol plus nitroglycerin (T + N + P), or CGP53353 plus nitroglycerine (T + N + C), respectively, for 24 h. All results are expressed as mean ± S.E.M., *n* = 7, *P < 0.05 compared with Con., T + N, T + P, T + C and T + N + C, *P* < 0.01 compared with Con., T + P, T + C and T + N + C.

We previously found that PKC-*β*_2_ activation played a critical role in TNF-*α* induced oxidative stress in endothelial cells [27], and further study have shown that gP91phox but not p22phox played an important role in TNF-*α* induced ROS production and HUVECs apoptosis [15]. Therefore, our present study measured the protein expression of p-PKC-*β*_2_ and gP91phox, one of the membrane subunits of NADPH oxidase, which catalyzes the generation of superoxide and is the major source of ROS in cardiovascular system [28]. As shown in Figure 5, the protein expressions of p-PKC-*β*_2_ and gP91phox were significantly increased in HUVECs subjected to TNF-*α* stimulation as compared to those of control group, which were prevented by the treatment of propofol or CGP53353. Addition of nitroglycerine further increased the protein expression of p-PKC-*β*_2_ and gP91phox, which was neutralized by propofol treatment but reversed by CGP53353 treatment.

## 4. Discussion

In the present study, we examined the protective effects of propofol against TNF-*α* induced toxicity in human umbilical vein endothelial cells in the presence or absence of nitrate tolerance. We demonstrated that propofol inhibited or prevented the adverse effects of TNF-*α* stimulation in the cultured endothelial cells. Furthermore, our results demonstrated that chronic treatment with nitroglycerine further exacerbated TNF-*α* induced cell toxicity by promoting PKC-*β*_2_ activation, with subsequently increased activation of NADPH oxidase, and ultimately neutralized the protective effects of propofol. This is the first study showing the role of PKC-*β*_2_ activation in nitroglycerin induced nitrate tolerance, which compromises the protective effects of propofol in endothelial cells subjected to TNF-*α* stimulation.

Endothelial dysfunction is implicated in a variety of cardiovascular diseases, such as hypercholesterolemia, atherosclerosis, hypertension, diabetes, and heart failure (see for review [29]). A relationship has been suggested to exist between inflammation and endothelial dysfunction [30]. TNF-*α*, one of the most important proinflammatory cytokines, is well known to increase ROS production in the endothelium and subsequently induce endothelial dysfunction [31]. This is well demonstrated by our present study showing that TNF-*α* resulted in a significant increase of LDH release and cell apoptosis, accompanied with increased superoxide and NOx production, elevated levels of the lipid peroxidation product MDA, and increased production of nitrotyrosine, a nitration product formed by peroxynitrite-mediated nitration of protein tyrosine residues. All these changes except NOx production were suppressed or prevented by propofol, an anesthetic with demonstrated antioxidant properties [9]. However, the precise mechanisms by which propofol attenuates TNF-*α* induced oxidative stress and endothelial dysfunction are not clear.

Endothelial NADPH oxidase is a major source of superoxide in blood vessels and is implicated in the oxidative stress accompanying various vascular diseases [32, 33]. NADPH oxidase contains two membrane-bound subunits gp91phox (Nox2) and p22phox and cytoplasmic subunits such as p47phox, p67phox, and a low-molecular-weight G protein (rac 1 and rac 2) [34]. Many protein kinase pathways have been involved in the regulation of NADPH oxidase activation, among which the PKC family seems to play an important role in this process [35]. PKC-*β* activation has been shown to play important or critical roles in NADPH oxidase activation [36, 37]. Interestingly, PKC-*β*_2_ is preferably upregulated in failing human hearts [38], which is accompanied with increased levels of TNF-*α* production [39] and NADPH oxidase activation [40]. Therefore, PKC-*β*_2_ and NADPH oxidase interplay may play critical roles in mediating cellular damage in situations associated with increased TNF-*α* production, such as AMI, heart failure, and diabetes, as well as during cardiac surgery using cardiopulmonary bypass. In the present study, propofol prevented TNF-*α* induced overexpression of p-PKC-*β*_2_ and gP91phox in endothelial cells. Of interest, the selective inhibitor of PKC-*β*_2_ CGP53353 has the similar effects as propofol. Therefore, we assumed that propofol preserves endothelial cells though inhibits PKC-*β*_2_ activation signal pathway, including inhibition of NADPH oxidase.

Although there are reports on PKC involvement upon NADPH oxidase activation after TNF-*α* stimulation in cultured HUVECs [8], especially in nitrate tolerance condition, the major or specific PKC isoform that is involved and the precise regulation mechanism remain unknown. Our previous study has demonstrated that PKC-*β*_2_ but not PKC-*δ* isoform pathway activation played dominant role in ROS production in this context [15]. However, a new finding in the present study showed that PKC-*β*_2_ activation was involved in NADPH oxidase activation in the condition of nitrate tolerance, a well known phenomenon that the clinical or hemodynamic response to organic nitrates (such as nitroglycerin, L-arginine) is attenuated or abolished after prolonged, continuous, or high dose nitrate treatment (see for review [2]). In the present study, supplementation of nitroglycerine further increased the apoptosis of endothelial cells and the activation of PKC-*β*_2_ induced by TNF-*α* stimulation, accompanied with enhanced levels of gP91phox and ROS production, which were reversed by the selective inhibition of PKC-*β*_2_ with CGP53353. This suggests that excessive activation of PKC-*β*_2_ and subsequent activation of NADPH oxidase paly a critical role in nitrate tolerance induced adverse effects. Of interest, propofol treatment reversed the increased levels of superoxide, MDA, nitrotyrosine, and the elevated protein expression of PKC-*β*_2_ and gP91phox, as well as LDH release and cell apoptosis in the endothelial cells after TNF-*α* stimulation. In the presence of nitroglycerine administration, however, propofol attenuated but not completely prevented these changes induced by TNF-*α* stimulation. This means that chronic treatment with nitroglycerin neutralized the protective effects of propofol.

In summary, the results from the present study indicate that nitrate tolerance further exacerbated TNF-*α* induced human vascular endothelial cell injury, as well as increased ROS production by PKC-*β*_2_ dependent activation of endothelial NADPH oxidase, and that the protective effects of propofol were compromised by nitroglycerine administration in experimental settings that are associated with persistent TNF-*α* stimulation. Further studies need to be performed in endothelial cell with deficit of the targeted kinase enzyme derived from gene knockout animals or gene silenced with specific antisenses to confirm the findings of the current study.

## References

[1] Nossaman VE, Nossaman BD, Kadowitz PJ (2010). Nitrates and nitrites in the treatment of ischemic cardiac disease. *Cardiology in Review*.

[2] Munzel T, Daiber A, Gori T (2013). More answers to the still unresolved question of nitrate tolerance. *European Heart Journal*.

[3] Münzel T, Daiber A, Mülsch A (2005). Explaining the phenomenon of nitrate tolerance. *Circulation Research*.

[4] Knorr M, Hausding M, Kröller-Schuhmacher S (2011). Nitroglycerin-induced endothelial dysfunction and tolerance involve adverse phosphorylation and S-glutathionylation of endothelial nitric oxide synthase: beneficial effects of therapy with the AT1 receptor blocker telmisartan. *Arteriosclerosis, Thrombosis, and Vascular Biology*.

[5] Parker JD (2004). Nitrate tolerance, oxidative stress, and mitochondrial function: another worrisome chapter on the effects of organic nitrates. *The Journal of Clinical Investigation*.

[6] Daiber A, Wenzel P, Oelze M, Münzel T (2008). New insights into bioactivation of organic nitrates, nitrate tolerance and cross-tolerance. *Clinical Research in Cardiology*.

[7] Scarabelli T, Stephanou A, Rayment N (2001). Apoptosis of endothelial cells precedes myocyte cell apoptosis in ischemia/reperfusion injury. *Circulation*.

[8] Li D, Yang B, Mehta JL (1999). Tumor necrosis factor-*α* enhances hypoxia-reoxygenation-mediated apoptosis in cultured human coronary artery endothelial cells: critical role of protein kinase C. *Cardiovascular Research*.

[9] Vasileiou I, Xanthos T, Koudouna E (2009). Propofol: a review of its non-anaesthetic effects. *European Journal of Pharmacology*.

[10] Ozkan D, Akkaya T, Yalcindag A (2013). Propofol sedation in total knee replacement : effects on oxidative stress and ischemia-reperfusion damage. *Der Anaesthesist*.

[11] Huang Z, Zhong X, Irwin MG (2011). Synergy of isoflurane preconditioning and propofol postconditioning reduces myocardial reperfusion injury in patients. *Clinical Science*.

[12] Liu K-X, Chen S-Q, Huang W-Q, Li Y-S, Irwin MG, Xia Z (2008). Propofol pretreatment reduces ceramide production and attenuates intestinal mucosal apoptosis induced by intestinal ischemia/reperfusion in rats. *Anesthesia and Analgesia*.

[13] Luo T, Xia Z, Ansley DM (2005). Propofol dose-dependently reduces tumor necrosis factor-*α*-induced human umbilical vein endothelial cell apoptosis: effects on Bcl-2 and bax expression and nitric oxide generation. *Anesthesia and Analgesia*.

[14] Xia Z, Luo T, Liu H-M (2010). L-arginine enhances nitrative stress and exacerbates tumor necrosis factor-*α* toxicity to human endothelial cells in culture: prevention by propofol. *Journal of Cardiovascular Pharmacology*.

[15] Deng B, Xie S, Wang J, Xia Z, Nie R (2012). Inhibition of protein kinase C *β*(2) prevents tumor necrosis factor-*α*-induced apoptosis and oxidative stress in endothelial cells: the role of NADPH oxidase subunits. *Journal of Vascular Research*.

[16] Wickley PJ, Shiga T, Murray PA, Damron DS (2006). Propofol decreases myofilament Ca2+ sensitivity via a protein kinase C-, nitric oxide synthase-dependent pathway in diabetic cardiomyocytes. *Anesthesiology*.

[17] Wickley PJ, Ding X, Murray PA, Damron DS (2006). Propofol-induced activation of protein kinase C isoforms in adult rat ventricular myocytes. *Anesthesiology*.

[18] Yu J, Kakutani T, Mizumoto K, Hasegawa A, Hatano Y (2006). Propofol inhibits phorbol 12, 13-dibutyrate-induced, protein kinase C-mediated contraction of rat aortic smooth muscle. *Acta Anaesthesiologica Scandinavica*.

[19] Kakiuchi-Kiyota S, Crabbs TA, Arnold LL (2013). Evaluation of expression profiles of hematopoietic stem cell, endothelial cell, and myeloid cell antigens in spontaneous and chemically induced hemangiosarcomas and hemangiomas in mice. *Toxicologic Pathology*.

[20] Zhang G-G, Shi R-Z, Jiang D-J (2008). Involvement of the endothelial DDAH/ADMA pathway in nitroglycerin tolerance: the role of ALDH-2. *Life Sciences*.

[21] Kaesemeyer WH, Ogonowski AA, Jin L, Caldwell RB, Caldwell RW (2000). Endothelial nitric oxide synthase is a site of superoxide synthesis in endothelial cells treated with glyceryl trinitrate. *British Journal of Pharmacology*.

[22] Lei S, Li H, Xu J (2013). Hyperglycemia-induced PKCbeta2 activation induces diastolic cardiac dysfunction in diabetic rats by impairing caveolin-3 expression and Akt/eNOS signaling. *Diabetes*.

[23] Xia Z-Y, Gao J, Ancharaz AK, Liu K-X, Xia Z, Luo T (2010). Ischaemic post-conditioning protects lung from ischaemia-reperfusion injury by up-regulation of haeme oxygenase-1. *Injury*.

[24] Xu B, Gao X, Xu J (2011). Ischemic postconditioning attenuates lung reperfusion injury and reduces systemic proinflammatory cytokine release via heme oxygenase 1. *Journal of Surgical Research*.

[25] Li Y-L, Gao L, Zucker IH, Schultz HD (2007). NADPH oxidase-derived superoxide anion mediates angiotensin II-enhanced carotid body chemoreceptor sensitivity in heart failure rabbits. *Cardiovascular Research*.

[26] Li J-M, Shah AM (2003). Mechanism of endothelial cell NADPH oxidase activation by angiotensin II. Role of the p47^phox^ subunit. *The Journal of Biological Chemistry*.

[27] Wang F, Liu H-M, Irwin MG (2009). Role of protein kinase C *β*2 activation in TNF-*α*-induced human vascular endothelial cell apoptosis. *Canadian Journal of Physiology and Pharmacology*.

[28] Lei S, Liu Y, Liu H, Yu H, Wang H, Xia Z (2012). Effects of N-acetylcysteine on nicotinamide dinucleotide phosphate oxidase activation and antioxidant status in heart, lung, liver and kidney in streptozotocin-induced diabetic rats. *Yonsei Medical Journal*.

[29] Cai H, Harrison DG (2000). Endothelial dysfunction in cardiovascular diseases: the role of oxidant stress. *Circulation Research*.

[30] Halaris A (2013). Inflammation, heart disease, and depression. *Current Psychiatry Reports*.

[31] Li J-M, Mullen AM, Yun S (2002). Essential role of the NADPH oxidase subunit p47^phox^ in endothelial cell superoxide production in response to phorbol ester and tumor necrosis factor-*α*. *Circulation Research*.

[32] Guzik TJ, West NE, Black E (2000). Vascular superoxide production by NAD(P)H oxidase: association with endothelial dysfunction and clinical risk factors. *Circulation Research*.

[33] Selemidis S, Dusting GJ, Peshavariya H, Kemp-Harper BK, Drummond GR (2007). Nitric oxide suppresses NADPH oxidase-dependent superoxide production by S-nitrosylation in human endothelial cells. *Cardiovascular Research*.

[34] Lyle AN, Griendling KK (2006). Modulation of vascular smooth muscle signaling by reactive oxygen species. *Physiology*.

[35] Inoguchi T, Nawata H (2005). NAD(P)H oxidase activation: a potential target mechanism for diabetic vascular complications, progressive *β*-cell dysfunction and metabolic syndrome. *Current Drug Targets*.

[36] Kitada M, Koya D, Sugimoto T (2003). Translocation of glomerular p47^phox^ and p67^phox^ by protein kinase C-*β* activation is required for oxidative stress in diabetic nephropathy. *Diabetes*.

[37] Dekker LV, Leitges M, Altschuler G (2000). Protein kinase C-*β* contributes to NADPH oxidase activation in neutrophils. *Biochemical Journal*.

[38] Noguchi T, Hünlich M, Camp PC (2004). Thin filament-based modulation of contractile performance in human heart failure. *Circulation*.

[39] Satoh M, Ishikawa Y, Itoh T, Minami Y, Takahashi Y, Nakamura M (2008). The expression of TNF-*α* converting enzyme at the site of ruptured plaques in patients with acute myocardial infarction. *European Journal of Clinical Investigation*.

[40] Nediani C, Borchi E, Giordano C (2007). NADPH oxidase-dependent redox signaling in human heart failure: relationship between the left and right ventricle. *Journal of Molecular and Cellular Cardiology*.

